# Subcutaneous Recurrences of Thyroid Cancer After Conventional Transcervical Thyroidectomy: A Case Report

**DOI:** 10.3389/fsurg.2020.586106

**Published:** 2020-11-05

**Authors:** Xubin Dong, Shihui Lv, Xiaohua Zhang, Quan Li

**Affiliations:** Department of Thyroid and Breast Surgery, The First Affiliated Hospital of Wenzhou Medical University, Wenzhou, China

**Keywords:** thyroid cancer, follicular variant of papillary thyroid cancer, subcutaneous implantation metastasis, ultrasound, computed tomography, imaging features

## Abstract

Metastatic subcutaneous implantation of the follicular variant of papillary thyroid cancer is very rare. We present a 62-year-old woman with a history of follicular variant of papillary thyroid cancer, who developed multiple asymptomatic subcutaneous nodules, after 5 years of initial thyroidectomy. Eventually, the subcutaneous nodules were diagnosed as tumor recurrence and completely excised. She has reportedly lived for more than 1 year, without signs of disease progression or recurrence. This case emphasizes the need for surgeons to take into account the tumor-free concept during the operation, and to a great extent prevent the occurrence of implantation recurrence.

## Background

Thyroid cancer is the world's most prevalent endocrine malignancy in the world, with most patients having a favorable prognosis (1). The most common pathological type of thyroid cancer is papillary thyroid cancer (PTC), which accounts for about 80% of all thyroid cancer cases (2). The second common type of thyroid cancer is follicular thyroid cancer (FTC), with a prevalence of 10–15% (3). The major subtype of PTC is the follicular variant of papillary thyroid cancer (FVPTC), which was first introduced in 1953 by Crile and Hazard as the alveolar variant of papillary thyroid cancer (4). Microscopically, FVPTC is made up almost entirely of cell-lined follicles with nuclear properties of papillary cancer cells. The prognosis of FVPTC falls between the classical PTC and FTC (5). Metastasis of FVPTC cutaneous implantation following thyroidectomy is a rare phenomenon with few reported cases.

This report describes a rare case in which a patient diagnosed with infiltrative FVPTC had subcutaneous implantation metastasis at the anterior neck and clavicle after 5 years of hemithyroidectomy with cervical incisions.

## Case Presentation

A 62-year-old woman was presented to the First Affiliated Hospital of Wenzhou Medical University, in November 2018 for palpable masses in the right neck for a week. Five years ago, in addition to the right hemithyroidectomy, she was treated in a local hospital for thyroid nodules with routine ipsilateral stage VI lymph node dissection. The subsequent histopathologic analysis revealed papillary thyroid cancer follicular variant (FVPTC). After the operation, the patient took a daily dose of levothyroxine tablets (100 μg).

**Figure 1:**
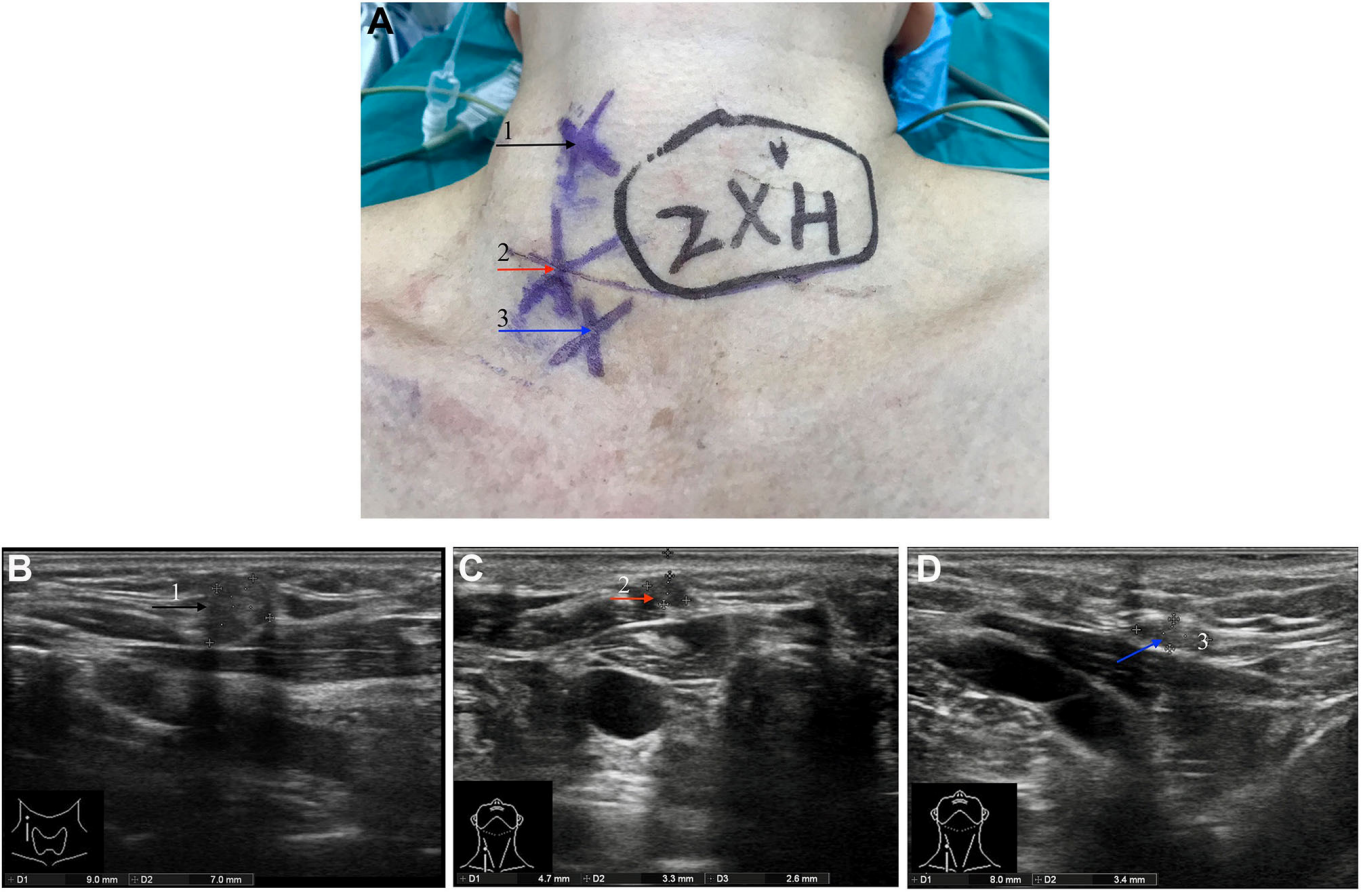
Pre-operative physical examination and ultrasound examination. **(A)** Three subcutaneous palpable nodules at the right anterior neck and the right supraclavicular. The neck ultrasound showed two hypoechoic nodules with a size of about 9^*^7^*^9 mm (F) and 5^*^3^*^7 mm (G) subcutaneously at the right anterior neck. A hypoechoic nodule with a size of about 8^*^4^*^6 mm (H) was observed at the right supraclavicular. Three nodules are annotated with black, red, and blue arrows, respectively, and corresponding the nodules indicated by the arrow in **(A)**.

**Figure 2:**
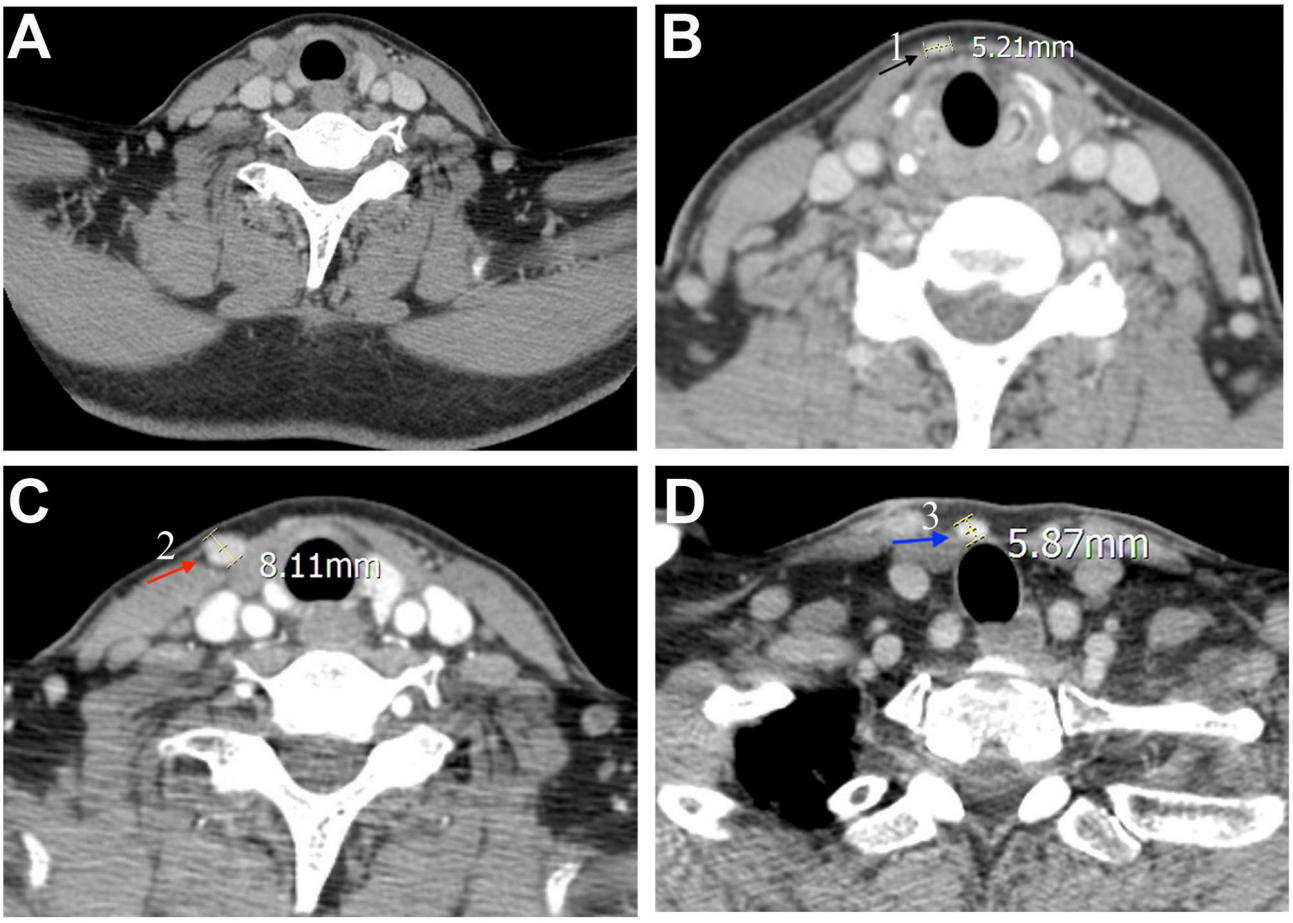
Pre-operative computed tomography findings. The neck enhanced computed tomography scans were observed **(A)** a general image of the neck, and **(B–D)** features of three nodules. The three nodules are annotated with black, red, and blue arrows, respectively, and corresponding nodules indicated by the arrow in Figure 1A.

In November 2018, the patient discovered multiple palpable masses in the right neck near the scar from the first operation and came to our hospital for further examination. Physical examination revealed several palpable nodules with a maximal size of about 1^*^1 cm located within the scar site of the previous surgery (Figure 1A). Neck ultrasonography revealed the presence of three hypoechoic nodules with a maximum size of 9^*^7^*^9 mm subcutaneously, at the right anterior neck and the right supraclavicular, characterized by irregular edges, irregular internal echoes, and intramodular blood flow (Figures 1B–D). We performed a fine-needle aspiration biopsy of one of the nodules to explain the histopathology of the nodules. The histologic findings suggested thyroid cancer infiltration or metastasis, and we recommended further surgical treatment, which she accepted. A preoperative enhanced computed tomography scan showed increased subcutaneous soft tissue mass in the right neck and multiple enlarged lymph nodes on both sides of the neck (Figures 2A–D). However, the thyroglobulin was normal, and the thyroid autoantibody tests were negative.

**Figure 3:**
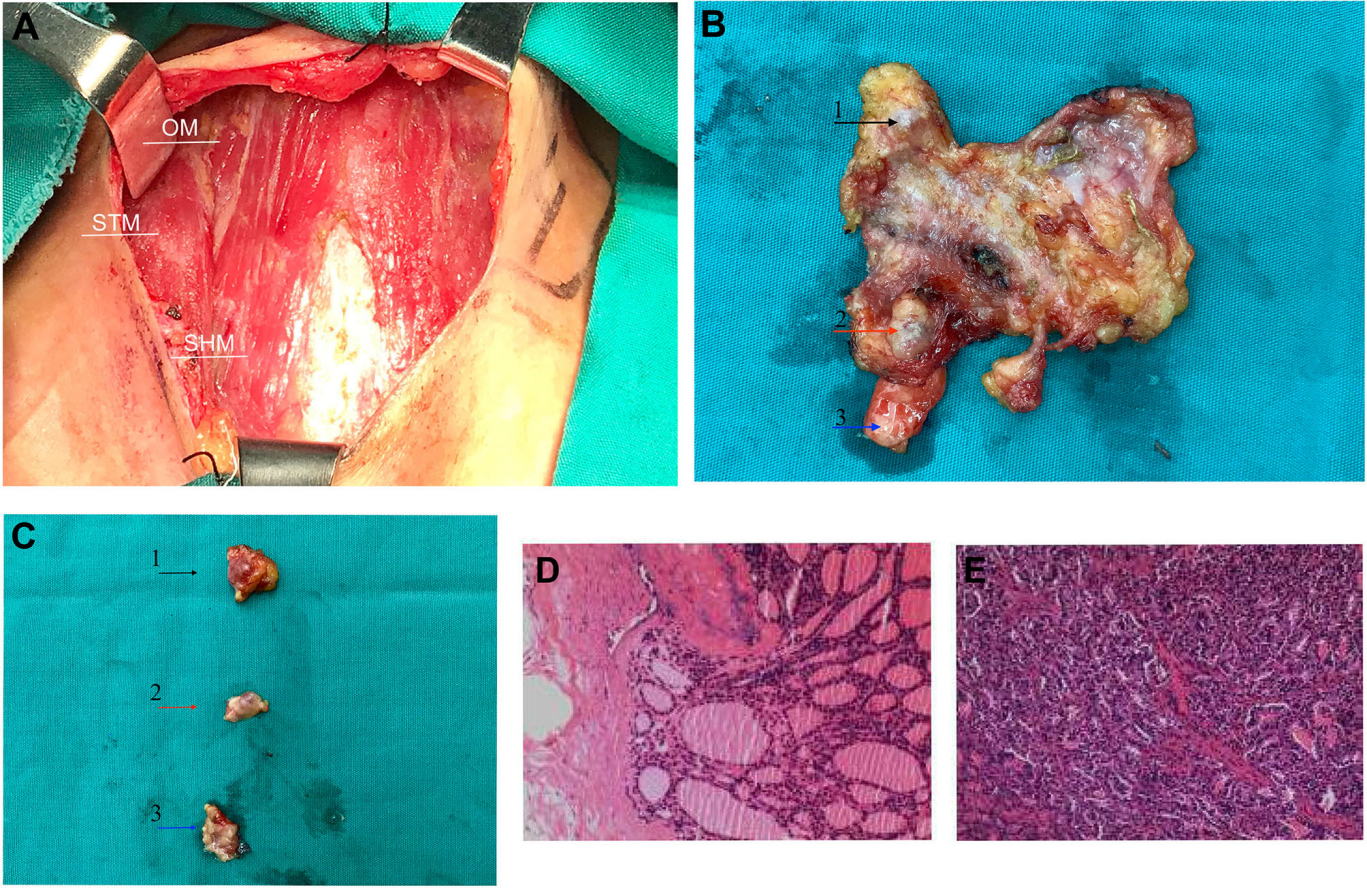
During surgery and post-operative image features. **(A)** The thyroid was incised, and the cervical fascia exposed intraoperatively. **(B)** The specimen was resected during surgery, and the arrows indicate the three palpable nodes. **(C)** Three removed nodules were presented individually, with a maximum size of 8^*^8 mm. Three nodules are annotated with black, red, and blue arrows, respectively, and corresponding nodules indicated by the arrow in Figure 1A. **(D,E)** Postoperative histopathology revealed subcutaneous nodules of the neck as metastatic carcinoma of follicular variant of papillary thyroid carcinoma. OM, omohyoid muscles; STM, sternothyroid muscle; SHM, sternohyoid muscle.

Following the completion of the preoperative examination and removal of the contraindications, right lateral (levels III/IV) neck dissection with wide excision of subcutaneous tumor tissue in addition to residual thyroidectomy was performed under general anesthesia. During the operation, we completely removed the superficial layer and nodules of the subcutaneous cervical fascia, the thyroid cartilage, the superior margin of the clavicle, the bilateral layer of the sternocleidomastoid muscle, and the anterior cervical fascia (Figures 3A,B). Three nodules with a maximum size of 8^*^8 mm were visible in the right subcutaneous and subclavian epithelium of the anterior neck, with a relatively hard texture (Figure 3C). Histopathology revealed the presence of thyroid tissue in the latissimus muscle, while the subcutaneous nodules were considered as infiltration or metastasis of FVPTC. Microscopic examination revealed atypical irregular follicular cells with ovoid nuclei, nuclear overlap, and nuclear grooves without papillary structures, as well as surrounding fibrous tissue reaction (Figures 3D,E). However, no metastatic lymph node was observed. The surgical operations were successfully performed, and the patient recovered well. After surgery, the patient received radioactive iodine therapy and was regularly monitored. She continued with the daily levothyroxine tablets (200 μg) dose and her condition is currently stable, with no signs of disease progression.

## Discussion

In recent years, FVPTC has accounted for almost one-third of all PTCs (6). However, the clinical characteristics of FVPTC remain controversial. Previous studies reported that the progressive and chronic FVPTC disease prevalence is higher than that of classical variants of papillary thyroid cancer (CVPTC). Other reports demonstrated that FVPTC has a lower incidence of lymph node and distant metastases, as well as a higher overall survival rate than CVPTC (7). The lung is the most common location of distant FVPTC metastasis, followed by the bones and the brain, with the latter having a poor prognosis (8). Although thyroid cancer rarely metastasizes to the skin, previous cases of FTC and PTC metastasis to the thyroidectomy scar have been reported (9, 10). Subcutaneous metastasis of FVPTC is still rare. Koller et al. (11) reviewed many cases of subcutaneous thyroid cancer metastasis and found that FVPTC is more prevalent than CVPTC, although the majority of metastasis sites were the scalp.

Several subcutaneous tumor metastasis mechanisms, including direct extension, hematogenous and lymphatic spread, and intraoperative implantation of shed tumor cells, have been postulated (12). The most probable pathogenesis of surgical scar metastasis could be intraoperative tumor cell implantation. In this case, we described the clinical history of a patient with subcutaneous implantation metastasis from FVPTC. We conclude that implantation metastasis is more likely than spontaneous metastasis in this case. First, the tumors recurred near the first surgical incision. Second, the pathologic diagnosis of the recurrent tumors was similar to that of the primary tumor. Third, recurrent tumors had no accompanying lymphatic or neurovascular tissue.

Subcutaneous metastases are associated with tumor cell dissemination and have a poor prognosis (13). However, a previous study reported that, in most cases, the prognosis of thyroid cancer with subcutaneous implantation metastases after FNAB is not impaired as seedings can be surgically resected without recurrence (14). Nevertheless, the clinical characteristics and prognosis of FVPTC with skin implant metastasis cannot be extrapolated, which needs further exploration.

In summary, preventing the spread of tumor cells during the operation is crucial. Factors related to meticulous surgical operation contribute to local implantation metastasis, with the contamination from inadequate tumor handling of the tumor being the main factor (15). Therefore, to control the contamination of benign tissue by viable malignant cells during surgery, the surgeon should adhere to the tumor-free principle during operation and should consider the risk of implantation metastasis following the surgery. Besides, subcutaneous metastases may be variable and typically asymptomatic in clinical presentation and are always discovered incidentally (16). Clinicopathology and immunohistochemistry, especially for poorly differentiated tumors, play an important role in the identification of suspicious skin lesions. In the treatment of cancer patients, clinicians should carefully examine skin involvement and pay attention to every skin nodule, even after a long asymptomatic period.

## Conclusions

In the present report, we describe a patient suffering from subcutaneous implantation metastasis of FVPTC, 5 years after hemithyroidectomy. We propose that surgeons should prevent the spread and contamination of tumor cells during surgery and attach importance to the identification of any skin lesion in patients with a history of FVPTC, so that the diagnosis is not delayed.

## Data Availability Statement

The original contributions presented in the study are included in the article/supplementary material, further inquiries can be directed to the corresponding author/s.

## Ethics Statement

The studies involving human participants were reviewed and approved by institutional review board of the First Affiliated Hospital of Wenzhou Medical University (Approval No. 2012-57). The patients/participants provided their written informed consent to participate in this study. Written informed consent was obtained from the individual(s) for the publication of any potentially identifiable images or data included in this article.

## Author Contributions

All authors have read and approved the final manuscript. XD performed the data acquisition and prepared the figures. XD and SL prepared the manuscript and followed up the patient. XZ and QL have been involved in the surgery and management of the patient. QL designed the report. All authors contributed to the article and approved the submitted version.

## Conflict of Interest

The authors declare that the research was conducted in the absence of any commercial or financial relationships that could be construed as a potential conflict of interest.
